# 5-Aminosalicylic acid inhibits stem cell function in human adenoma-derived cells: implications for chemoprophylaxis in colorectal tumorigenesis

**DOI:** 10.1038/s41416-021-01354-5

**Published:** 2021-03-30

**Authors:** Steven William Dixon, Tracey Jane Collard, Eleanor May Harrisdotter Mortensson, Danny Nigel Legge, Adam Christian Chambers, Alexander Greenhough, Tom Julian Creed, Ann Caroline Williams

**Affiliations:** 1 Cellular and Molecular Medicine, Biomedical Sciences Building, University of Bristol, Bristol, BS8 1TD UK; 2 Bristol Royal Infirmary, University Hospitals Bristol and Weston NHS Foundation Trust, Bristol, BS2 8HW UK; 3 Department of Applied Sciences, Centre for Research in Biosciences, University of the West of England, Bristol, BS16 1QY UK

**Keywords:** Colorectal cancer, Preclinical research, Cancer prevention

## Abstract

**Background:**

Most colorectal cancers (CRC) arise sporadically from precursor lesions: colonic polyps. Polyp resection prevents progression to CRC. Risk of future polyps is proportional to the number and size of polyps detected at screening, allowing identification of high-risk individuals who may benefit from effective chemoprophylaxis. We aimed to investigate the potential of 5-aminosalicylic acid (5-ASA), a medication used in the treatment of ulcerative colitis, as a possible preventative agent for sporadic CRC.

**Methods:**

Human colorectal adenoma (PC/AA/C1, S/AN/C1 and S/RG/C2), transformed adenoma PC/AA/C1/SB10 and carcinoma cell lines (LS174T and SW620) were treated with 5-ASA. The effect on growth in two- and three-dimensional (3D) culture, β-catenin transcriptional activity and on cancer stemness properties of the cells were investigated.

**Results:**

5-ASA was shown, in vitro, to inhibit the growth of adenoma cells and suppress β-catenin transcriptional activity. Downregulation of β-catenin was found to repress expression of stem cell marker LGR5 (leucine-rich G protein-coupled receptor-5) and functionally suppress stemness in human adenoma and carcinoma cells using 3D models of tumorigenesis.

**Conclusions:**

5-ASA can suppress the cancer stem phenotype in adenoma-derived cells. Affordable and well-tolerated, 5-ASA is an outstanding candidate as a chemoprophylactic medication to reduce the risk of colorectal polyps and CRC in those at high risk.

## Background

Colorectal cancer (CRC) is one of the most common malignancies worldwide and, despite advances in treatment, is the second most common cause for cancer-related mortality.^1^ Of concern, the incidence of CRC appears to be rising in adults under the age of 50 years.^2,3^ CRC derives from pre-neoplastic precursor lesions—polyps—which can be resected before malignant transformation. Accordingly, many countries have employed national screening programmes that allow identification of polyps and cancers at early stages when they are more amenable to curative treatment. These screening programmes also allow identification of individuals at high risk of CRC: in the recent joint British Society of Gastroenterology/Association of Coloproctology of Great Britain and Ireland/Public Health England guidelines, individuals with high-risk findings are offered further surveillance colonoscopy (high-risk findings are defined by the presence of either (a) two or more polyps (excluding diminutive hyperplastic rectal polyps 1–5 mm), of which one polyp is ≥10 mm or (b) ≥5 polyps of any size).^4^ Despite this, there is currently no chemoprophylaxis that is offered to reduce the risk of further polyps or CRC for these individuals.

Aspirin has demonstrated promise as a chemoprophylactic drug in this context; several clinical trials have reported a reduction in adenoma number with regular aspirin use.^5,6^ Most recently, the seAFOod (Systematic Evaluation of Aspirin and Fish Oil) polyp prevention trial recruited patients with high-risk endoscopic findings from the English Bowel Cancer Screening Programme and reported reduced number of polyps in the aspirin-treated group at follow-up colonoscopy (although the adenoma detection rate was not significantly reduced).^7^ Further, clinical trials have demonstrated reduced colorectal polyp burden in patients with the hereditary cancer syndrome Familial Adenomatous Polyposis and halved the incidence of CRC in patients with Lynch syndrome following aspirin use.^8^ This has resulted in the recent recommendation by the National Institute of Clinical Excellence endorsing the prescription of prophylactic aspirin for Lynch syndrome mutation carriers.^9^ However, aspirin is associated with an increased risk of bleeding, exemplified by the findings of two recent, large randomised controlled trials.^10,11^ The ARRIVE (Aspirin to Reduce Risk of Initial Vascular Events) trial reported that 100 mg aspirin daily doubled the risk of gastrointestinal bleeding (hazard ratio (HR) 2.11, 95% confidence interval (CI) 1.36–3.28),^10^ similar to that reported in the ASPREE trial (HR 1.87, 95% CI 1.32–2.66).^11^ The ASPREE (Aspirin in Reducing Events in the Elderly) trial also reported that the risk of intracranial bleeding was increased by 50% in healthy adults over 70 years old (HR 1.5, 95% CI 1.11–2.02).^11^ Consequently, aspirin may not be a suitable chemoprophylactic drug in all patients and certainly the benefit and harm needs to be carefully assessed before use.^12^

5-Aminosalicylic acid (5-ASA) is a non-steroidal anti-inflammatory drug structurally similar to aspirin which is commonly prescribed to induce and maintain remission in chronic idiopathic inflammatory bowel disease (IBD). Conventional subclassification of IBD distinguishes two phenotypically categorised conditions: ulcerative colitis (UC) and Crohn’s disease (CD). UC and CD are both associated with an increased risk of CRC: the so-called colitis-associated cancer (CAC).^13,14^

Although a complete understanding of the anti-inflammatory mechanisms of 5-ASA is lacking, existing data imply that 5-ASA has efficacy in suppressing multiple pro-inflammatory pathways: 5-ASA has been demonstrated to antagonise several pro-inflammatory mediators including interferon-γ,^15^ tumour necrosis factor-α^15,16^ and nuclear factor-κB,^16,17^ which may be, at least in part, due to agonism of peroxisome proliferator-activated receptor-γ (PPARγ).^18^ Information from epidemiology studies is limited, but early observational data indicated that 5-ASA reduced the risk of CAC,^19^ although a 2012 meta-analysis reported a protective effect in clinic-based studies with no effect in population-based studies.^20^ However, the two most-recent meta-analyses by Qiu et al.^21^ and Bonovas et al.^22^ reported dose-dependent protective effects of oral mesalazine across a range of study designs, including pooled analysed of population-based studies, in UC. Accordingly, the European Crohn’s and Colitis Organisation have recommended lifelong oral 5-ASA as chemoprophylaxis against CAC.^23^ Importantly, 5-ASA is well-tolerated, is not associated with increased risk of bleeding and is affordable for health providers. However, it remains unknown whether 5-ASA confers a reduced risk of developing sporadic CRC.

The mechanisms underpinning the apparent antineoplastic activity of 5-ASA in CAC have not been fully elucidated, but existing data from models of CRC have suggested that 5-ASA may suppress Wnt/β-catenin through multiple mechanisms including those implicated in its anti-inflammatory role including induction of PPARγ;^18,24^ suppression of the cyclo-oxygenase-2/prostaglandin E_2_ (PGE_2_) axis;^25^ post-translational modification of the β-catenin phosphatase protein phosphatase 2A.^26^ 5-ASA may also promote membranous sequestration of β-catenin through N-glycosylation of and membranous translocation of E-cadherin;^27^ negative regulation of the serine/threonine protein kinase PAK1;^28^ upregulation of µ-protocadherin.^29^ Importantly, mutations resulting in upregulated Wnt/β-catenin signalling are among the first observed in colorectal adenomas and have been demonstrated as being sufficient for early adenoma formation.^30^ Evidence that 5-ASA inhibits the β-catenin signalling in adenomas comes from immunohistochemical analysis as part of the German 5-ASA Polyp Prevention Trial: Munding et al.^31^ reported reduced expression of β-catenin in adenomas from patients taking 1 g 5-ASA/day. However, to date, these results have not been validated either in vitro or in vivo in human adenoma, and the effect of 5-ASA on adenoma growth is unknown. Further, given that Wnt/β-catenin signalling is important for the maintenance of the colonic stem compartment,^32^ we hypothesised that suppression of dysregulated Wnt/β-catenin may suppress the stem phenotype, which, crucially, may prevent adenoma formation and progression in sporadic disease.

While the effect of 5-ASA on the growth of carcinoma-derived cells in vitro has been described,^33–38^ no such data exist for cells derived from colonic adenomas. In this study, we aimed to establish the effect of 5-ASA on Wnt/β-catenin and stem cell phenotype in human adenoma using adenoma-derived cells in two-dimensional (2D) and three-dimensional (3D) models of tumorigenesis in order to understand whether 5-ASA may be an effective chemoprophylactic drug for individuals at high risk of sporadic CRC.

## Methods

### Cell lines and culture

The colorectal adenoma-derived cell lines PC/AA/C1, S/AN/C1 and S/RG/C2 and the transformed adenoma-derived cell line PC/AA/C1/SB10 used in these experiments were established in this laboratory, their derivation and characterisation have been previously described.^39–41^ Growth medium was Dulbecco’s modified Eagle’s medium (DMEM) (Gibco; Thermo Fisher Scientific, MA, USA) supplemented with 20% foetal bovine serum (FBS), 1 µg/mL hydrocortisone sodium succinate (Sigma-Aldrich; Merck, MO, USA), 0.2 U/mL insulin (Sigma-Aldrich; Merck, MO, USA), 2 mM glutamine (Gibco; Thermo Fisher Scientific, MA, USA), 100 U/mL penicillin and 100 μg/mL streptomycin (Gibco; Thermo Fisher Scientific, MA, USA). The CRC-derived cell lines LS174T and SW620 were obtained from American Type Culture Collection (Rockville, MD, USA) were cultured in DMEM supplemented with 10% FBS, 2 mM glutamine, 100 U/mL penicillin and 100 U/mL streptomycin. All cell lines were routinely assessed for microbial contamination (including mycoplasma) and characterised using an in-house panel of cellular and molecular markers to check that cell lines have not been cross-contaminated (every 3–6 months; data not shown). Stocks were securely catalogued and stored, and passage numbers strictly adhered to prevent phenotypic drift.

### Treatments

5-ASA (Sigma-Aldrich; Merck, MO, USA) was dissolved in culture media, pH balanced to 7.35–7.45, sterile-filtered and supplemented with HEPES buffer solution 1 M (Sigma-Aldrich; Merck, MO, USA) (20 µL per 1 mL 5-ASA solution).

All cell lines were seeded into 25 cm^2^ tissue culture flasks (T25; Corning, NY, USA): all adenoma-derived cell lines were seeded at 2 × 10^6^ cells/flask (except PC/AA/C1, seeded at 4 × 10^6^ cells/flask), transformed adenoma and CRC-derived cell lines at 1 × 10^6^ cells/flask. Seeding densities were calculated so that all cell lines were 70% confluent when treated with 5-ASA. After 72 h, the culture media were replaced by 20–40 mM 5-ASA/culture media solution. At 24, 48, and 72 h after the addition of 5-ASA, floating cells were collected, attached cells were trypsinised and both were counted in triplicate for each condition.

### Immunoblotting

Whole-cell lysates were prepared in situ, on ice and analysed by western blotting as previously described^42^ using antibodies to the following: AXIN-2 (2151, Cell Signaling, MA, USA, 1:1000), β-catenin (9587, Cell Signaling, MA, USA, 1:5000), active-β-catenin (05-665, Millipore, Sigma, MA, USA, 1:1000), c-MYC (SC-40, Santa Cruz Biotechnology, CA, USA, 1:200), LEF-1 (2230, Cell Signaling, MA, USA, 1:1000) and LGR5 (Ab75850, Abcam, Cambridge, UK, 1:1000). Equal loading was confirmed using β-actin (A5316, Sigma-Aldrich, Merck, MO, USA. 1:1000) or α-tubulin (T9026, Sigma-Aldrich, Merck, MO, USA. 1:10,000).

### TOPflash reporter assay

Cells were treated with 5-ASA 24 h after transfection with TOPflash/FOPflash and SV40-Renilla plasmids as previously described^43^ using the Promega Dual Luciferase Reporter Assay System (Promega, WI, USA) according to the manufacturer’s instructions. FOPflash reporter with mutated TCF consensus sites was used to control for non-specific output. Luminescence was measured at 560 nm using a Modulus luminometer (Turner Biosciences, CA, USA).

### RNA interference

Cells were transfected using Lipofectamine RNAiMAX (Invitrogen, Thermo Fisher Scientific, MA, USA), according to the manufacturer’s protocol, with small interfering RNAs (siRNAs, final concentration 50 nM; Dharmacon, Horizon Discovery, Cambridge, UK) targeting LEF-1, or a negative control, for which four different siRNA sequences were pooled.^44^ Cells were incubated overnight at 37 °C before medium changing. Samples were prepared 72 h after transfection.

### Spheroid formation assay

Spheroids formed from adenoma- and carcinoma-derived cells were grown using an adapted protocol from the original Sato paper.^45^ Cells were resuspended in Matrigel (Corning, NY, USA) as a single-cell suspension and seeded into 24-well plates (Corning, NY, USA) as described previously.^46^ The Matrigel hemispheres were allowed to polymerise before being submerged in advanced DMEM:F12 (Gibco, Thermo Fisher Scientific, MA, USA) supplemented with 0.1% bovine serum albumin (Sigma-Aldrich, Merck, MO, USA), 2 mM glutamine (Gibco, Thermo Fisher Scientific, MA, USA), 10 mM HEPES (Sigma-Aldrich, Merck, MO, USA), 100 U/mL penicillin and 100 U/mL streptomycin (Gibco; Thermo Fisher Scientific, MA, USA), 1% N2 (Thermo Fisher Scientific, MA, USA), 2% B27 (Thermo Fisher Scientific, MA, USA) and 0.2% *N*-acetylcysteine (Sigma-Aldrich, Merck, MO, USA). For spheroid culture of PC/AA/C1 adenoma-derived cells, the spheroid medium was further supplemented with human epidermal growth factor (Peprotech, London, UK), 50 ng/mL. The culture media were refreshed twice weekly over the course of 21 days in culture. Wells were imaged as Z-stacks using a Leica DM16000 widefield microscope and LAS-X software (both Leica Microsystems, Wetzlar, Germany) on days 7, 11, 14, 18 and 21. Images acquired were analysed using MATLAB R2015a software (MathWorks, MA, USA).

### Quantitative PCR (qPCR)

Total RNA was extracted from spheroids using TRI-reagent (Sigma-Aldrich, Merck, MO, USA); a RNeasy mini kit (Qiagen, Hilden, Germany) was utilised according to the manufacturer’s protocol with an additional on-column DNase digestion step (RNase-Free DNase Set; Qiagen, Hilden, Germany). Complementary DNA synthesis was synthesised from 2 µg RNA, using the RNA-dependent DNA polymerase, Moloney murine leukaemia virus reverse transcriptase (Promega WI, USA). The samples were diluted to a final concentration of 10 ng/µL. Following optimisation of primers and ensuring the annealing temperature provided ~100% amplification efficiency per cycle (data not shown), qPCR was performed, as previously described,^47^ using SYBR Green PCR mix (Qiagen, Hilden, Germany) and the following Qiagen QuantiTect primers, LGR5 (cat. no. QT00027720) and CD133 (cat. no. QT00075586), with gene expression normalised interchangeably with both housekeeping genes TATA-binding protein (TBP; cat. no. QT00000721) or hypoxanthine phosphoribosyl transferase (HPRT; cat. no. QT00059066). Amplification data were analysed using MxPro software version 4.10 (Agilent Technologies, CA, USA).

### Statistical analysis

All statistical analysis was performed using GraphPad Prism software, student edition (GraphPad Software, California, USA). *P* values were determined using either one-sample *t* test or one-way analysis of variance testing with Bonferroni post-test. Results are expressed as mean values ± SEM or ±SD where specified.

## Results

### 5-ASA suppresses the growth of adenoma- and CRC-derived cells in vitro

**Fig. 1:**
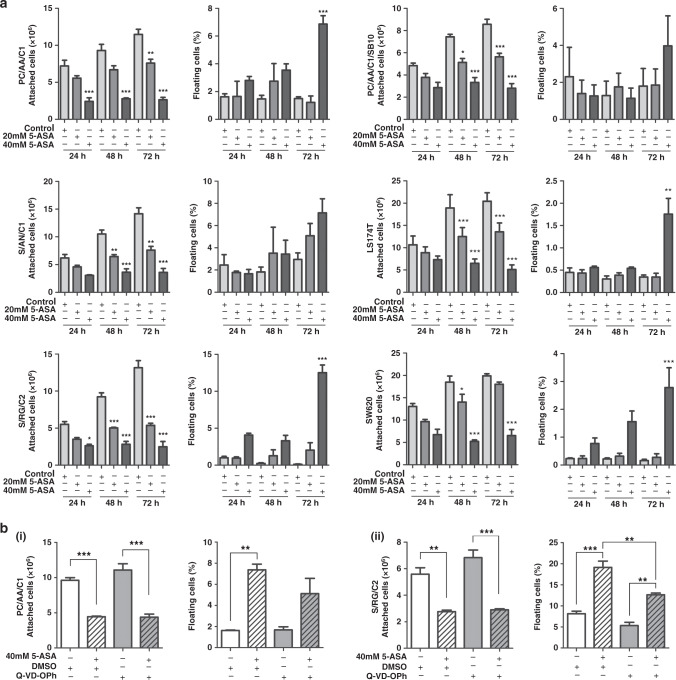
5-Aminosalicylic acid inhibits the growth of human colonic adenoma cells as well as carcinoma cell lines in two-dimensional culture. **a** Graphs show attached cell yield and the number of floating cells as a proportion of total cell yield of three adenoma-derived cell lines (PC/AA/C1, S/AN/C1 and S/RG/C2), left, transformed adenocarcinoma cells (PC/AA/C1/SB10) and two CRC-derived cell lines (LS174T and SW620), right, 24, 48 and 72 h after treatment with 20 and 40 mM 5-ASA. Mean ± SEM; *n* = 3. One-way ANOVA with Bonferroni post-test, *p < 0.05; **p < 0.01; ***p < 0.001. **b** Graphs show attached cell yield and percentage of floating cells of (i) PC/AA/C1 and (ii) S/RG/C2; after treatment with 40 mM 5-ASA and 10 µM Q-VD-Oph or DMSO, cells were harvested and counted at 72 h. Mean ± SEM; *n* = 3. One-way ANOVA with Bonferroni post-test, **p < 0.01; ***p < 0.001.

Three adenoma-derived cell lines (PC/AA/C1, S/AN/C1 and S/RG/C2) were seeded in T25 flasks for 72 h before treatment with 20 or 40 mM 5-ASA (consistent with concentrations used previously^33–38^) and the attached cell yield and floating cells counted after 24, 48 and 72 h (Fig. 1a). This experiment was also carried out in the transformed adenoma cell line PC/AA/C1/SB10 and two CRC cell lines (LS174T and SW620, Fig. 1a). 5-ASA inhibited the number of attached cells in all adenoma-derived cell lines treated with either 20 or 40 mM 5-ASA. Of interest, the adenoma-derived cell lines were as sensitive to 5-ASA treatment as the tumorigenic cell lines (Fig. 1a).

In two of the three adenoma-derived cell lines, there was a significant induction of floating cells 72 h after 40 mM 5-ASA treatment, indicative of cell death. However, the reduction in cell yield on treatment with 5-ASA could not be explained by induction of cell death alone; in support of this, blocking apoptosis with the pan-caspase inhibitor (Q-VD-OPh) did not rescue the reduction in cell yield on 40 mM 5-ASA treatment (Fig. 1b). Furthermore, although 5-ASA induced apoptosis in cancer-derived cells LS174T, blocking it also had no effect on the inhibition of cell yield when treated with 40 mM 5-ASA (Supplementary Figure 1A); this is consistent with 5-ASA inducing growth inhibition in the cancer cells as previously reported.^33–38^ Taken together, these results show that 5-ASA causes growth inhibition in both the colorectal adenoma- and carcinoma-derived cells.

### 5-ASA downregulates Wnt/β-catenin signalling in human adenoma and carcinoma cells

**Fig. 2:**
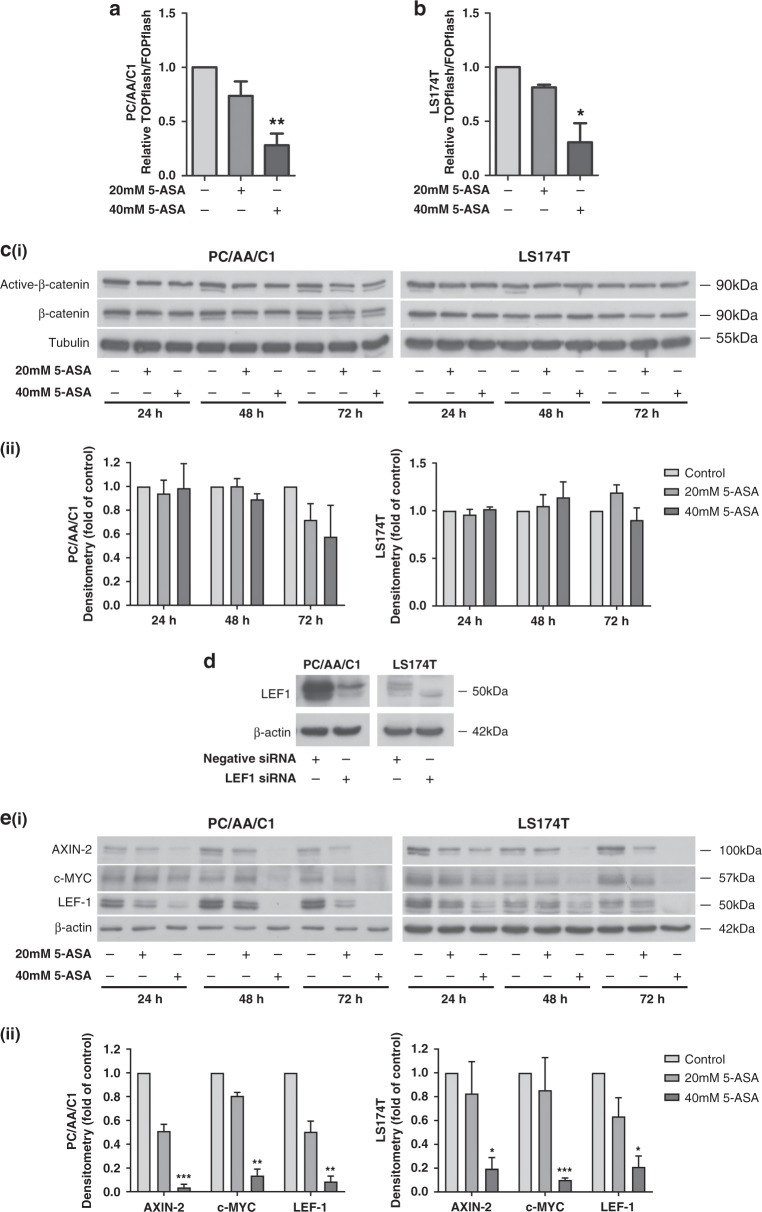
5-ASA suppresses β-catenin/TCF transcriptional activity. **a**, **b** TOPflash reporter assay at 24 h after 20 and 40 mM 5-ASA. **a** PC/AA/C1 adenoma and **b** LS174T CRC-derived cells. Mean ± SEM; *n* = 3; *p < 0.05; **p < 0.01 **c** (i) Western blots of PC/AA/C1 and LS174T at 24, 48 and 72 h after treatment with 20 and 40 mM 5-ASA showing expression of active dephosphorylated and total β-catenin protein; α-tubulin was used as the loading control. (ii) Densitometry graphs show the fold change of active dephosphorylated β-catenin protein as a ratio of total β-catenin expression over the 72-h period. Expression is normalised to the respective control. Data are presented as the mean ± SEM of three independent experiments. *n* = 3. One sample *t* test was used to determine statistical significance. **d** Western blot of LEF-1 expression in PC/AA/C1 and LS174T cells to determine the specificity of the LEF-1 antibody. The expression level of LEF-1 was measured by western blotting 72 h after transfection with a LEF-1 SMARTpool siRNA or negative control. The results are representative of three independent experiments. β-Actin was used as the loading control. **e** (i) Western blot showing PC/AA/C1 and LS174T cells after 24, 48 and 72 h after treatment with 20 and 40 mM 5-ASA. Wnt/β-catenin target proteins AXIN-2, c-MYC and LEF-1 are all downregulated with 5-ASA, with the most marked phenotype observed at 72 h. β-Actin was used as the loading control. (ii) Densitometry graphs show the expression change of AXIN-2, c-MYC and LEF-1 as a fold of the loading control at the 72-h timepoint. Expression is normalised to the respective control. Data are presented as the mean ± SEM of three independent experiments. *n* = 3. One sample *t* test was used to determine statistical significance. *p < 0.05; **p < 0.01; ***p < 0.001.

To measure the effect of 5-ASA on β-catenin/TCF-mediated transcription activity in adenoma-derived cells, we treated PC/AA/C1 (APC mutant) adenoma-derived cells with 5-ASA after transfection with TOPflash and FOPflash reporter plasmids and compared it to LS174T (β-catenin mutant) carcinoma-derived cells. These cell lines were chosen as representative of tumours with disrupted β-catenin signalling, important for the initiation of colorectal carcinogenesis. At 24 h treatment, 40 mM 5-ASA TOPflash activity was significantly suppressed (Fig. 2a, b, results for the transformed adenoma-derived cells are shown in Supplementary Figure 1B). A similar, but not statistically significant trend was observed for cells treated with 20 mM 5-ASA. Interestingly, total cellular β-catenin levels and active dephosphorylated β-catenin were unchanged on western blots after 5-ASA treatment in all cell lines (Fig. 2c and Supplementary Figure 2A, C). Next, we investigated β-catenin target expression after treatment with 5-ASA (Fig. 2e and Supplementary Figure 2B, D). Accordingly, known β-catenin-regulated proteins AXIN-2, c-MYC and LEF-1 were downregulated by 20 and 40 mM 5-ASA in both a dose- and time-dependent manner with the most marked effects observed at the higher dose at the 72 h time point (Fig. 2e and Supplementary Figure 2B, D).

### 5-ASA reversibly suppresses expression of the stem-marker LGR5 in colorectal adenoma and carcinoma cells

**Fig. 3:**
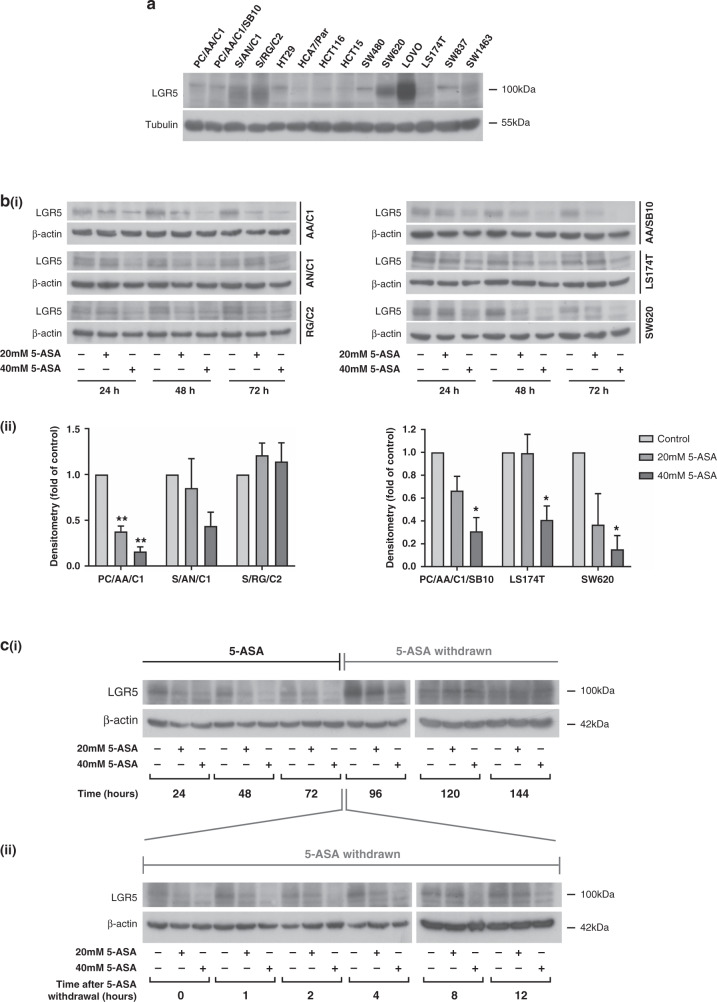
5-ASA suppresses the expression of the stem-marker LGR5. **a** Endogenous levels of LGR5 expression in a panel of colorectal adenoma- and carcinoma-derived cell lines. PC/AA/C1, S/AN/C1, S/RG/C2 colorectal cells, PC/AA/C1/SB10 transformed adenocarcinoma cells, HT29, HCA7, HCT116, HCT15, SW480, SW620, LOVO, LS174T colorectal adenocarcinoma cells and SW837 and SW1463 rectal adenocarcinoma cells were grown to ~70% confluence before collection of total protein for western blot analysis. α-Tubulin used as the loading control. **b** (i) Western blot analysis demonstrating downregulation of LGR5 in three adenomas (PC/AA/C1, S/AN/C1 and S/RG/C2), left, transformed adenocarcinoma cells (PC/AA/C1/SB10) and two CRC-derived cell lines (LS174T and SW620), right, 24, 48 and 72 h after treatment with 20 and 40 mM 5-ASA. β-Actin was used as a loading control. LGR5 is highly glycosylated^59^, leading to the different banding patterns seen in the different cell lines. (ii) Densitometry graphs show the expression change of LGR5 as a fold of the loading control at the 72h timepoint. Expression is normalised to the respective control. Data are presented as the mean ± SEM of three independent experiments. *n* = 3. One sample *t* test was used to determine statistical significance. *p < 0.05; **p < 0.01. **c** (i) Western blots of LGR5 expression in PC/AA/C1 adenoma cells demonstrating downregulation of LGR5 after commencing treatment with 20 and 40 mM 5-ASA, but a subsequent reversal of this regulation once 5-ASA was withdrawn. β-Actin was used as a loading control. (ii) Western blot analysis of LGR5 expression in the 12 h after stopping 5-ASA treatment. β-Actin was used as a loading control.

LGR5 (Leucine Rich Repeat Containing G Protein-Coupled Receptor 5) is an established marker of crypt-base stem cells.^48^ LGR5 expression is frequently expressed in adenoma and tumour metastases but expression in primary CRC is variable^49^ (Fig. 3a). Western blots show that 5-ASA suppresses expression of LGR5 in 2/3 adenomas, the transformed adenoma and both CRC-derived cells, over a 72-h period (Fig. 3b). Because LGR5 is a β-catenin-regulated gene, we hypothesised that the effect of 5-ASA would be reversible, important for the maintenance of tissue homeostasis in the surrounding colonic epithelium. To determine whether expression of LGR5 recovered on removal of 5-ASA, PC/AA/C1 adenoma cells were treated with 5-ASA for 72 h before washing the cells and culturing for a further 3 days without 5-ASA. Western blots demonstrated that re-expression of LGR5 was noted within 12 h of stopping 5-ASA treatment in PC/AA/C1 cells (Fig. 3c) with expression returning to baseline 48 h after stopping treatment.

### Low-dose 5-ASA reduces the ability of PC/AA/C1 cells to form spheroids

LGR5^+^ stem cells form spheroid structures when grown in extracellular matrix gels such as Matrigel, complete with differentiated colonic cells and hierarchical organisation as seen in the gastrointestinal tract in vivo.^45^ As such, the ability of cells to form spheroids from single-cell suspensions is considered an assay of stemness.^50^

**Fig. 4:**
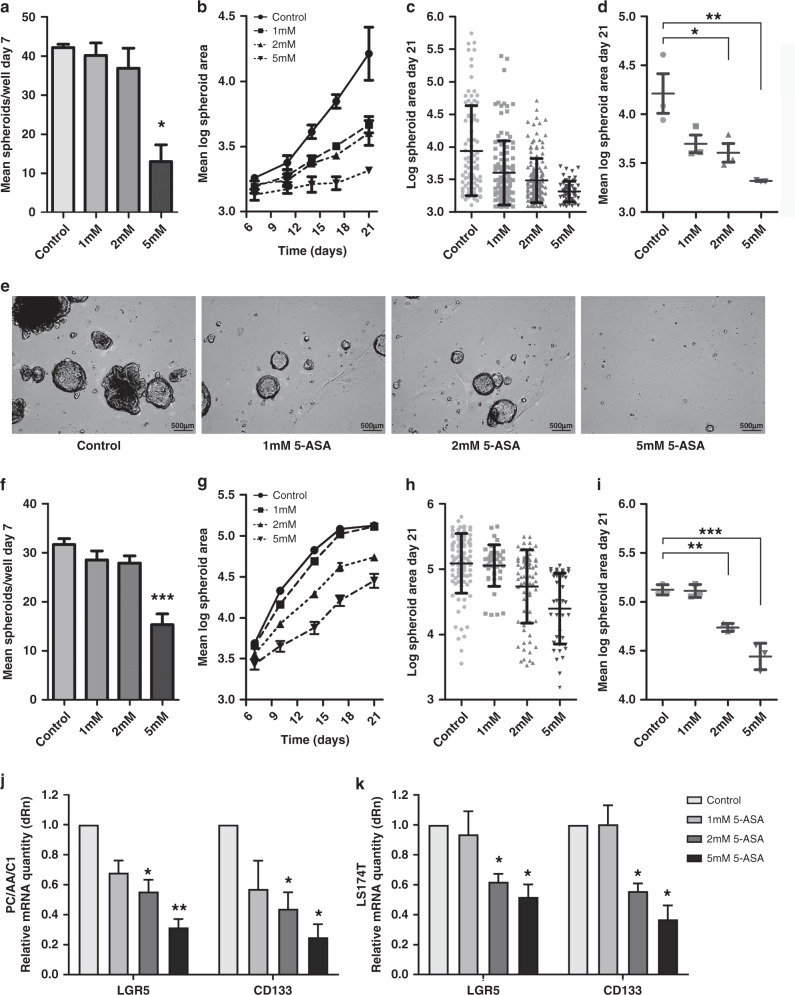
5-ASA reduces the ability of PC/AA/C1 adenoma and LS174T carcinoma cells to form spheroids. **a**–**e** PC/AA/C1 human adenoma-derived cells. **a** Mean number of spheroids in each well 7 days after culture. *n* = 3 ± SEM; *p < 0.05. **b** Mean log spheroid area over 21 days in culture. **c** Log spheroid area of spheroids at day 21, demonstrating distribution of spheroid size. *n* = 1 ± SD representative of *n* = 3. **d** Mean log spheroid area of spheroids at day 1. *n* = 3 ± SEM; *p < 0.05; **p < 0.01. **e** Representative images of PC/AA/C1-derived spheroids at day 21. Images acquired using Leica DM16000 microscope, ×5 lens with the Leica LAS-X software. Images were processed using MatLab software. **f**–**i** LS174T human carcinoma-derived cells. **f** Mean number of spheroids in each well 7 days after culture. *n* = 3 ± SEM; ***p < 0.001. **g** Mean log spheroid area over 21 days in culture. **h** Log spheroid area of spheroids at day 21, demonstrating distribution of spheroid size. *n* = 1 ± SD representative of *n* = 3. **i** Mean log spheroid area of spheroids at day 21. *n* = 3 ± SEM; **p < 0.01; ***p < 0.001. **j**–**k** Quantitative PCR (QPCR) mRNA analysis of LGR5 and CD133 gene expression. **j** PC/AA/C1- and **k** LS174T-derived spheroids after 21 days of treatment with 5-ASA. All mRNA values are normalised to the housekeeping genes *TBP* or *HPRT*. Data show relative mRNA quantity of LGR5 and CD133 presented as a fold change of the control, which itself was normalised to one. Data are presented as the mean of three independent experiments ± SEM; *n* = 3. One sample *t* test was used to determine statistical significance, *p < 0.05; **p < 0.01. dRn baseline-corrected normalised fluorescence.

To establish whether 5-ASA could inhibit stem cell function, PC/AA/C1 cells were seeded as a single-cell suspension into Matrigel. As PC/AA/C1 cells were more sensitive to 5-ASA in 3D culture than 2D culture, they were treated with 1–5 mM 5-ASA at the time of seeding. 5 mM 5-ASA treatment was sufficient to significantly block spheroid formation as well as growth of the adenoma-derived cells (Fig. 4); there were significantly fewer spheroids after 7 days in 3D culture (Fig. 4a). In addition, spheroid size analysis showed that 5-ASA treatment resulted in significantly smaller spheroids after 21 days in culture (Fig. 4b–e). Similar findings were noted for LS174T-derived CRC spheroids (Fig. 4f–i). Furthermore, the messenger RNA (mRNA) expression of stem cell-associated proteins LGR5 and CD133 was significantly decreased in the 2 and 5 mM treated PC/AA/C1 and LS174T cells (Fig. 4j, k).

**Fig. 5:**
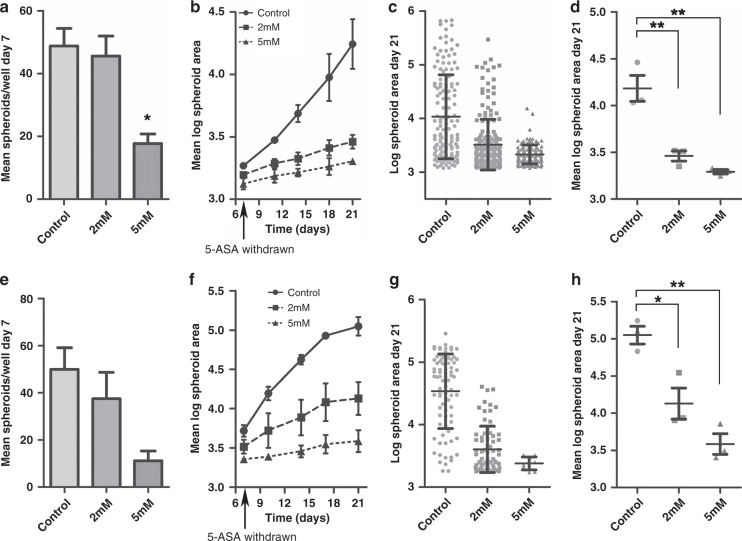
The growth inhibitory effect of 5-ASA on PC/AA/C1 adenoma- and LS174T carcinoma-derived spheroids is sustained for 14 days after treatment is stopped. **a**–**d** PC/AA/C1 human adenoma-derived cells. **a** Mean number of spheroids per well after 7 days in culture. *n* = 3 ± SEM; *p < 0.05. **b** Mean log spheroid area over 21 days in culture. 5-ASA was withdrawn from the culture media on day 7. *n* = 3 ± SEM. **c** Log spheroid area after 21 days in culture, demonstrating distribution of spheroid size. *n* = 1 ± SD (representative of *n* = 3). **d** Mean log spheroid area of spheroids at day 21. *n* = 3 ± SEM; **p < 0.01. **e**–**h** LS174T human carcinoma-derived cells. **e** Mean number of spheroids per well after 7 days in culture. *n* = 3 ± SEM. **f** Mean log spheroid area over 21 days in culture. 5-ASA was withdrawn from the culture media on day 7. *n* = 3 ± SEM. **g** Log spheroid area after 21 days in culture, demonstrating distribution of spheroid size. *n* = 1 ± SD (representative of *n* = 3). **h** Mean log spheroid area of spheroids at day 21. *n* = 3 ± SEM; *p < 0.05; **p < 0.01.

Importantly, when these experiments were repeated with 5-ASA removed from the culture media at day 7, the growth inhibitory effect on the PC/AA/C1 spheroids was sustained for a further 14 days in culture (Fig. 5a–d). Similar results were obtained for LS174T CRC-derived spheroids (Fig. 5e–h). This finding further suggests that 5-ASA suppresses the stem cell potential of the cells, as both the number and the growth of the spheroids was unable to fully recover to that of the untreated spheroids once the 5-ASA is removed.

## Discussion

The results presented here provide new insights into the effect of 5-ASA, an affordable and well-tolerated drug, on the growth and stemness potential of adenoma-derived cells in vitro. Important for use in a cancer prevention setting, this is the first report to document the effect of 5-ASA on adenoma-derived spheroids in 3D culture. 3D cell culture using spheroids is a useful model for studying stem function; this is exemplified by elegant work from the Sato group who generated a ‘library’ of spheroids derived from colorectal adenoma and carcinomas and demonstrated that not only did niche dependency decrease along the adenoma–carcinoma sequence, but that spheroids reproduced the histopathological grade and differentiation capacity of their parental tumours both in vitro and as xenografts.^51^ Our data demonstrated that 5-ASA consistently negatively regulated Wnt/β-catenin activity and target gene expression, directly antagonising a key signalling pathway of the colonic stem compartment. Further, for the first time, 5-ASA was demonstrated to negatively regulate expression of the stem cell marker LGR5 (and stem-associated protein CD133). In addition, by blocking the formation of adenoma-derived spheroids, 5-ASA was shown to functionally suppress stemness.

Targeting adenoma cells with stem cell properties is important because colorectal tumorigenesis is believed to be initiated and driven by a subpopulation of cells with properties of stemness—cancer stem cells—typified by asymmetric cell division and slow cell turnover making them resistant to traditional chemotherapeutics.^52^ Lineage-tracing experiments have demonstrated that LGR5^+^ cells act as stem cells in mouse adenoma and genetic tracing of LGR5^+^ clones in tumour xenografts derived from human CRC organoids have demonstrated that these cells have the ability to generate both differentiated cells and self-renew.^53^ Further, LGR5^+^ cells drive adenoma growth in mouse models^54^ and promote adenoma cell survival in human adenoma.^49^ Baker et al.^48^ demonstrated using in situ hybridisation that LGR5 expression is increased in adenomas with expression throughout the adenomatous gland, with a heterogeneous distribution and loss of stem hierarchy observed in normal mucosa. Because LGR5 is a ‘Wnt amplifier’^55,56^ expansion of LGR5^+^ cells may be a key step in allowing cells without permissive mutations in other pro-oncogenic signalling pathways to expand, driving the formation of adenomas. Thus, it is possible that suppression of LGR5 may suppress the stem potential of adenoma cells and may prevent adenoma formation. Importantly, suppression of β-catenin transcriptional activity and spheroid formation was demonstrated at 5-ASA concentrations that are achievable with available 5-ASA preparations: oral 5-ASA preparations equivalent to 2 g/day achieve luminal concentrations of 12–22.7 mM.^57^ This corresponds with observational epidemiological data suggesting that 5-ASA >1.2 g/day is protective against CAC.^21^

How relevant 5-ASA-mediated negative regulation of LGR5 is for established CRC remains less clear. While LGR5 is commonly expressed/overexpressed in adenomas,^48^ expression is frequently low/absent in CRCs before re-expression in metastatic deposits;^49^ indeed, LGR5^+^ cells appear to be important in metastatic progression.^58^ As summarised by Morgan et al.,^59^ there is abundant contradictory data on the role of LGR5 in CRC. This may be explained, at least in part, by plasticity exhibited by CSCs. Shimokawa et al.^53^ recently demonstrated that LGR5^+^ carcinoma cells differentiated into both LGR5^+^KRT20^−^ and LGR5−KRT20^+^ daughters, and that selective ablation of LGR5 (using a CRISPR-Cas9 system) resulted initially in tumour regression followed by re-expression of LGR5 and recovery of tumour growth. In this context, using 5-ASA to prevent the re-expression of LGR5 may not only prevent tumour formation and potentially recurrence after treatment but may also improve the efficacy of conventional therapies, improving the prognosis of patients with CRC. Importantly in the 3D cultures, it was possible to demonstrate that the growth inhibitory effect of 5-ASA on the PC/AA/C1 spheroids was sustained for a further 14 days in culture after removal of the drug, suggesting that continuous administration of 5-ASA may not be necessary for either chemoprophylaxis or therapy.

5-ASA is an affordable and well-tolerated drug with decades of clinical experience in the treatment of UC, making it an outstanding candidate as a chemoprophylactic agent for patients at risk of CRC. Perhaps, surprisingly, for a drug that has been known for some time to suppress β-catenin activity in CRC cells, there have been a lack of clinical trials to assess the efficacy of 5-ASA in the prevention of sporadic CRC. In targeting the stemness potential of adenoma-derived cells, results from this study provide new evidence to support the use of 5-ASA for the prevention of colorectal carcinogenesis. Taken together with evidence from other studies, including analysis of the effect of 5-ASA in patient samples,^31^ we believe that robust clinical trials are now required to understand whether these findings translate into a reduction in adenoma burden in at-risk individuals.

## Disclaimer

The work presented in this article is original research. This article has not been previously published and has not been submitted for publication elsewhere while under consideration.

## Supplementary information

Supplementary figures

## Data Availability

All data supporting the results are presented with results and in the figures.
