# Supplementary material for: 5-Aminosalicylic acid inhibits stem cell function in human adenoma-derived cells: implications for chemoprophylaxis in colorectal tumorigenesis
Source: Br J Cancer. 2021 Mar 30;124(12):1959–69. doi: 10.1038/s41416-021-01354-5 (PMC8184823; doi:10.1038/s41416-021-01354-5)
Supplement: Supplementary file 1 — Supplementary figures [file 41416_2021_1354_MOESM1_ESM.pptx]

## Slide 1
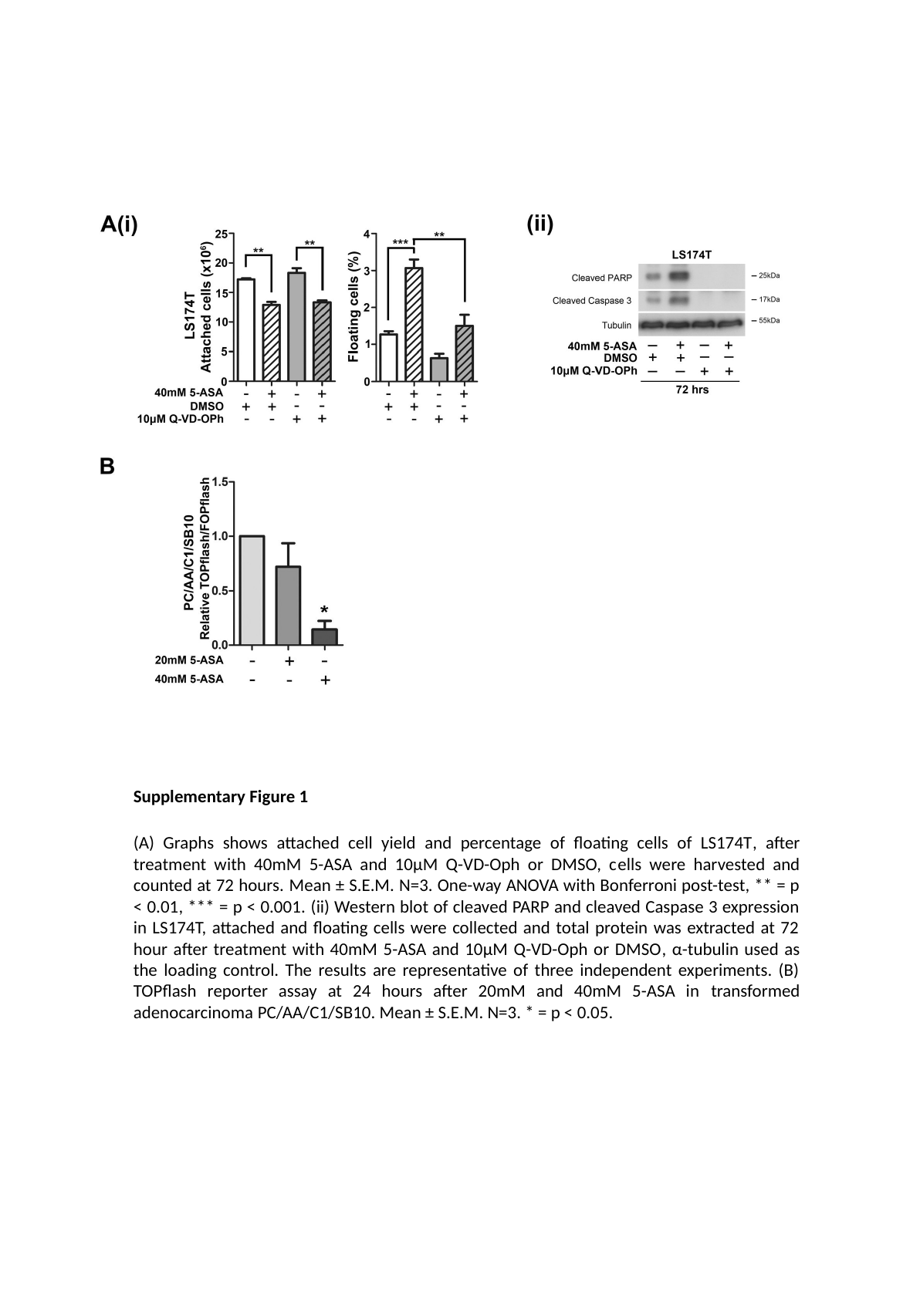

Supplementary Figure 1
(A) Graphs shows attached cell yield and percentage of floating cells of LS174T, after treatment with 40mM 5-ASA and 10µM Q-VD-Oph or DMSO, cells were harvested and counted at 72 hours. Mean ± S.E.M. N=3. One-way ANOVA with Bonferroni post-test, ** = p < 0.01, *** = p < 0.001. (ii) Western blot of cleaved PARP and cleaved Caspase 3 expression in LS174T, attached and floating cells were collected and total protein was extracted at 72 hour after treatment with 40mM 5-ASA and 10µM Q-VD-Oph or DMSO, α-tubulin used as the loading control. The results are representative of three independent experiments. (B) TOPflash reporter assay at 24 hours after 20mM and 40mM 5-ASA in transformed adenocarcinoma PC/AA/C1/SB10. Mean ± S.E.M. N=3. * = p < 0.05.

## Slide 2
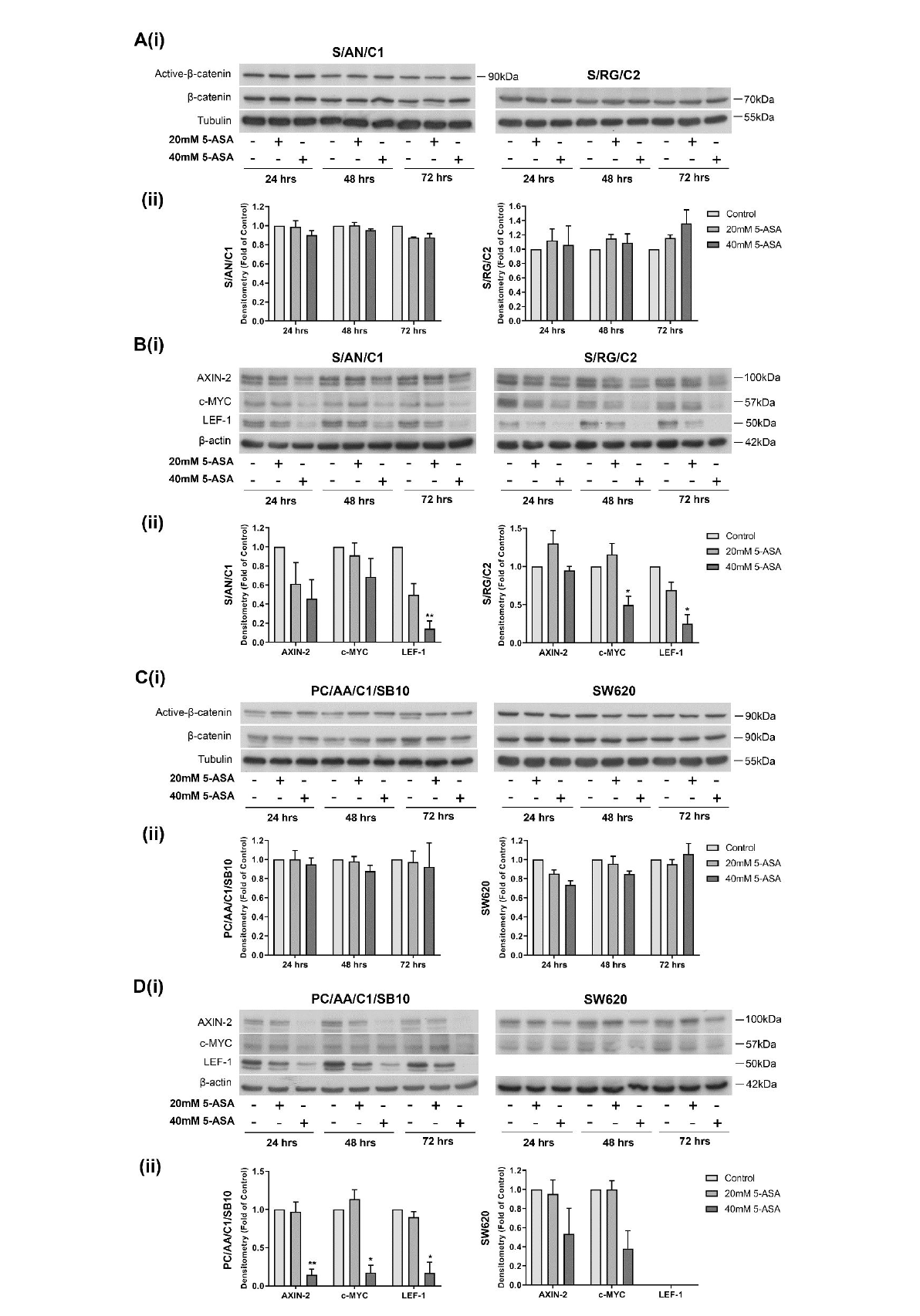

#

## Slide 3
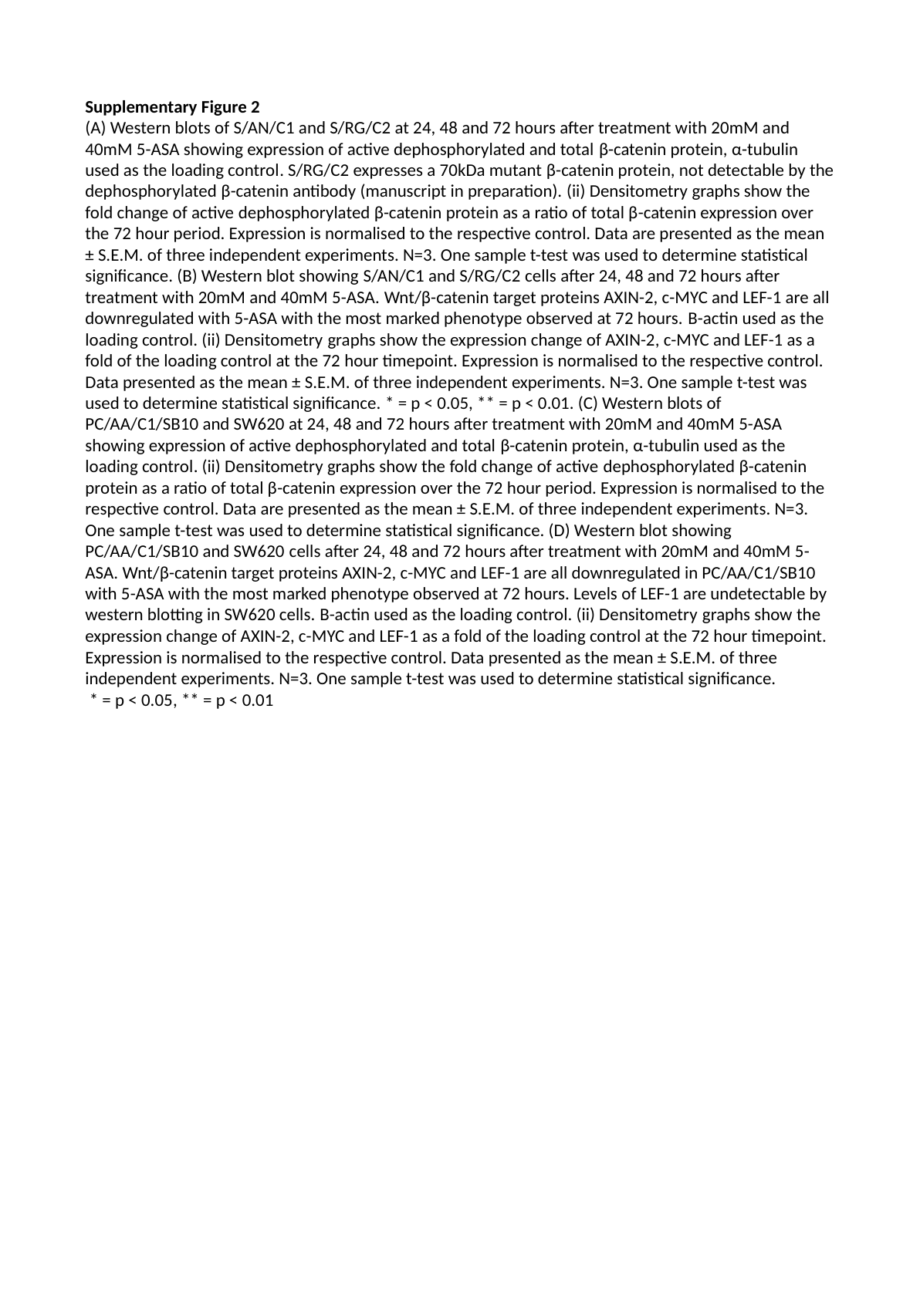

Supplementary Figure 2
(A) Western blots of S/AN/C1 and S/RG/C2 at 24, 48 and 72 hours after treatment with 20mM and 40mM 5-ASA showing expression of active dephosphorylated and total β-catenin protein, α-tubulin used as the loading control. S/RG/C2 expresses a 70kDa mutant β-catenin protein, not detectable by the dephosphorylated β-catenin antibody (manuscript in preparation). (ii) Densitometry graphs show the fold change of active dephosphorylated β-catenin protein as a ratio of total β-catenin expression over the 72 hour period. Expression is normalised to the respective control. Data are presented as the mean ± S.E.M. of three independent experiments. N=3. One sample t-test was used to determine statistical significance. (B) Western blot showing S/AN/C1 and S/RG/C2 cells after 24, 48 and 72 hours after treatment with 20mM and 40mM 5-ASA. Wnt/β-catenin target proteins AXIN-2, c-MYC and LEF-1 are all downregulated with 5-ASA with the most marked phenotype observed at 72 hours. Β-actin used as the loading control. (ii) Densitometry graphs show the expression change of AXIN-2, c-MYC and LEF-1 as a fold of the loading control at the 72 hour timepoint. Expression is normalised to the respective control. Data presented as the mean ± S.E.M. of three independent experiments. N=3. One sample t-test was used to determine statistical significance. * = p < 0.05, ** = p < 0.01. (C) Western blots of PC/AA/C1/SB10 and SW620 at 24, 48 and 72 hours after treatment with 20mM and 40mM 5-ASA showing expression of active dephosphorylated and total β-catenin protein, α-tubulin used as the loading control. (ii) Densitometry graphs show the fold change of active dephosphorylated β-catenin protein as a ratio of total β-catenin expression over the 72 hour period. Expression is normalised to the respective control. Data are presented as the mean ± S.E.M. of three independent experiments. N=3. One sample t-test was used to determine statistical significance. (D) Western blot showing PC/AA/C1/SB10 and SW620 cells after 24, 48 and 72 hours after treatment with 20mM and 40mM 5-ASA. Wnt/β-catenin target proteins AXIN-2, c-MYC and LEF-1 are all downregulated in PC/AA/C1/SB10 with 5-ASA with the most marked phenotype observed at 72 hours. Levels of LEF-1 are undetectable by western blotting in SW620 cells. Β-actin used as the loading control. (ii) Densitometry graphs show the expression change of AXIN-2, c-MYC and LEF-1 as a fold of the loading control at the 72 hour timepoint. Expression is normalised to the respective control. Data presented as the mean ± S.E.M. of three independent experiments. N=3. One sample t-test was used to determine statistical significance.
 * = p < 0.05, ** = p < 0.01
